# A Novel Genetic Variant in Long Non-coding RNA Gene *NEXN-AS1* is Associated with Risk of Lung Cancer

**DOI:** 10.1038/srep34234

**Published:** 2016-10-07

**Authors:** Hua Yuan, Hongliang Liu, Zhensheng Liu, Kouros Owzar, Younghun Han, Li Su, Yongyue Wei, Rayjean J. Hung, John McLaughlin, Yonathan Brhane, Paul Brennan, Heike Bickeboeller, Albert Rosenberger, Richard S. Houlston, Neil Caporaso, Maria Teresa Landi, Joachim Heinrich, Angela Risch, David C. Christiani, Zeynep H. Gümüş, Robert J. Klein, Christopher I. Amos, Qingyi Wei

**Affiliations:** 1 Collaborative Innovation Center For Cancer Personalized Medicine, Nanjing Medical University; Department of Oral and Maxillofacial Surgery, Affiliated Hospital of Stomatology, Nanjing Medical University, Nanjing, China; 2 Department of Medicine, Duke University School of Medicine, Durham, NC, USA; 3 Duke Cancer Institute, Duke University Medical Center, Durham, NC, USA; 4 Department of Biostatistics and Bioinformatics, Duke University Medical Center, Durham, NC, USA; 5 Community and Family Medicine, Geisel School of Medicine, Dartmouth College, Hanover, NC, USA; 6 Massachusetts General Hospital, Boston, Massachusetts, USA; 7 Department of Environmental Health, Harvard School of Public Health, Boston, Massachusetts, USA; 8 Lunenfeld-Tanenbaum Research Institute of Mount Sinai Hospital, Toronto, Ontario, Canada; 9 Public Health Ontario, Toronto, Ontario, Canada; 10 Genetic Epidemiology Group, International Agency for Research on Cancer (IARC), Lyon, France; 11 Department of Genetic Epidemiology, University Medical Center, Georg-August-University Göttingen, Göttingen, Germany; 12 Division of Genetics and Epidemiology, The Institute of Cancer Research, London, United Kingdom; 13 Genetic Epidemiology Branch, Division of Cancer Epidemiology and Genetics, National Cancer Institute, National Institutes of Health, Bethesda, MD, USA; 14 Helmholtz Centre Munich, German Research Centre for Environmental Health, Institute of Epidemiology I, Neuherberg, Germany; 15 Institute and Outpatient Clinic for Occupational, Social and Environmental Medicine, University Hospital Munich, Ludwig Maximilian University Munich, Munich, Germany; 16 Department of Molecular Biology, University of Salzburg, Salzburg, Austria; 17 Department of Epigenomics and Cancer Risk Factors, DKFZ-German Cancer Research Center, Heidelberg, Germany; 18 Translational Lung Research Center Heidelberg (TLRC-H), Member of the German Center for Lung Research (DZL), Heidelberg, Germany; 19 Department of Genetics and Genomic Sciences, Icahn Institute for Genomics and Multiscale Biology, Icahn School of Medicine at Mount Sinai, New York, USA

## Abstract

Lung cancer etiology is multifactorial, and growing evidence has indicated that long non-coding RNAs (lncRNAs) are important players in lung carcinogenesis. We performed a large-scale meta-analysis of 690,564 SNPs in 15,531 autosomal lncRNAs by using datasets from six previously published genome-wide association studies (GWASs) from the Transdisciplinary Research in Cancer of the Lung (TRICL) consortium in populations of European ancestry. Previously unreported significant SNPs (*P* value < 1 × 10^−7^) were further validated in two additional independent lung cancer GWAS datasets from Harvard University and deCODE. In the final meta-analysis of all eight GWAS datasets with 17,153 cases and 239,337 controls, a novel risk SNP rs114020893 in the lncRNA *NEXN-AS1* region at 1p31.1 remained statistically significant (odds ratio = 1.17; 95% confidence interval = 1.11–1.24; *P* = 8.31 × 10^−9^). In further *in silico* analysis, rs114020893 was predicted to change the secondary structure of the lncRNA. Our finding indicates that SNP rs114020893 of *NEXN-AS1* at 1p31.1 may contribute to lung cancer susceptibility.

Lung cancer is one of the most common cancers worldwide. In the United States, it is estimated that 221,200 new lung cancer cases will occur in 2015^1^. Despite of much devoted research effort in the treatment for lung cancer in recent decades, it remains the leading cause of cancer deaths among males worldwide and the leading cause of cancer deaths among females in more developed countries^2^. Although smoking has been confirmed to be the most common risk factor for lung cancer, only about one-tenth of the smokers develop lung cancer in their lifetimes^3^, which suggests that other factors play important roles in lung carcinogenesis^4^. Over the past few years, genome-wide association studies (GWASs) of lung cancer have identified multiple loci associated with lung cancer risk^5^,^6^,^7^,^8^,^9^,^10^. Several of those loci (e.g., 6p21, 5p15, 3p28 and 15q25) have been validated in multiple studies^11^,^12^. These findings have greatly advanced our knowledge of the genetic basis of lung cancer in humans.

Although much attention has been focused on the expression of protein-coding genes, accumulating evidence suggests that non-coding RNAs (ncRNAs) have specialized regulatory and processing functions. For example, genetic variants of microRNAs play important roles in cancers^13^,^14^,^15^. To date, however, little is known about the association between genetic variation of long non-coding RNAs (lncRNAs) and lung cancer risk. LncRNAs are a new class of transcripts that were recently discovered, which are pervasively transcribed in the genome and critical regulators of the epigenome^16^. Emerging studies have demonstrated the major biological roles of lncRNAs in a variety of processes that have an impact on carcinogenesis, embryonic development, or metabolism^17^. Recently, SNPs in several lncRNA genes previously identified to be involved in cancer development have been reported to be associated with cancer risk, e.g. rs7763881 in the hepatocellular cancer-related *HULC* gene^18^ and rs920778 in the gastric cancer-related *HOTAIR* gene^19^. These results provide some evidence for the important roles of lncRNA SNPs in carcinogenesis.

Currently, little is known about the associations between genetic variants of lncRNAs and lung cancer risk. In the present study, we re-visited several published GWASs and evaluated the effects of lncRNA SNPs on lung cancer risk by using a large-scale meta-analysis of six previously published lung cancer GWAS datasets from the Transdisciplinary Research in Cancer of the Lung (TRICL) consortium and two additional GWAS datasets of independent Caucasian populations from Harvard University and Icelandic lung cancer study^8^.

## Results

**Figure 1:**
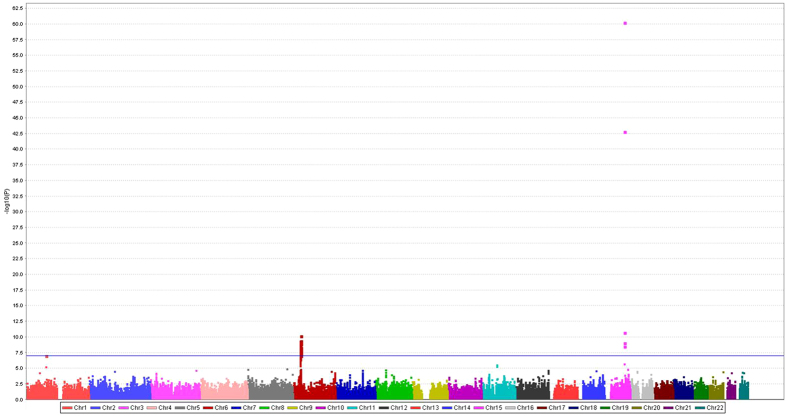
Manhattan plot of associations between SNPs of the lncRNA genes and risk of lung cancer. There were 59 SNPs with a *P* < 1 × 10^−7^.

The combined dataset of six previously published GWAS datasets used for the initial analysis (discovery) consisted of 12,160 cases and 16,838 controls of European ancestry. The initially identified associations between SNPs in lncRNAs and lung cancer risk are shown in Fig. 1. In brief, the meta-analysis of 690,564 SNPs in lncRNAs from the TRICL consortium showed that 59 SNPs were associated with lung cancer risk with a *P* value < 1 × 10^−7^, and no heterogeneity among these GWAS datasets was noted, except for one SNP of rs35031105 (Supplementary Table 1). Of these 59 SNPs, 53 from 11 lncRNAs were located at the lung cancer risk-related loci 6p21.33 and 6p22.1 that have been reported previously^6^,^20^. The other five SNPs from three lncRNAs were located in 15q25.1 that was also reported by multiple studies^5^,^10^,^21^. Therefore, we focused on the remaining previously unreported SNP rs114020893 located in lncRNA *NEXN-AS1* (also known as C1orf118) on chromosome 1p31.1 for the further analysis.

**Figure 2:**
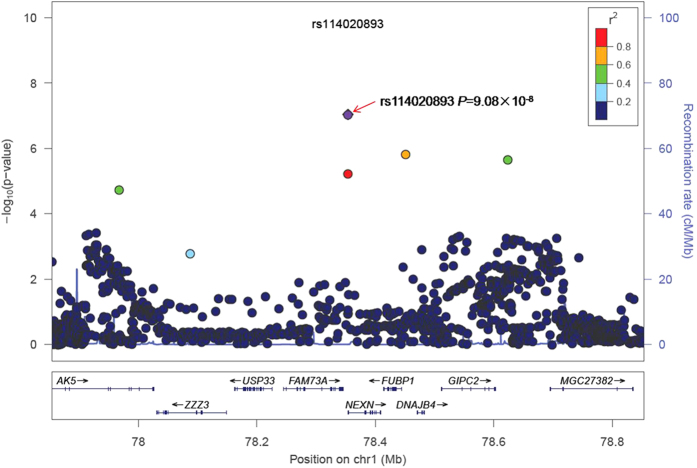
Regional association plot of rs114020893. The left-hand Y-axis shows the *P*-value of individual SNPs, which is plotted as −log10(*P*) against chromosomal base-pair position.The right-hand Y-axis shows the recombination rate estimated from the HapMap CEU population.

For the purposes of illustration, the forest plot of the meta-analysis of rs114020893 using the six GWAS datasets is presented in Supplementary Figure 1. For the rs114020893 C variant allele in the GWAS datasets from the Institute of Cancer Research (ICR), the MD Anderson Cancer Center (MDACC), the International Agency for Research on Cancer (IARC), the National Cancer Institute (NCI), the Samuel Lunenfeld Research Institute study (Toronto), and the German Lung Cancer Study (GLC)^21^, the allele frequencies were 0.098, 0.092, 0.072, 0.084, 0.073 and 0.066, respectively; the additive odds ratio (OR) were 1.20, 1.42, 1.41, 1.18, 1.28 and 1.11, respectively; and its risk effect was significant in the first four datasets with a larger number of observations. There was no heterogeneity observed among these datasets, with *I*^2^ of 0 and the Q-test *P* value of 0.672 in the meta-analysis. In the combined results, the per-unit increase of the C allele was associated with1.23-fold increase of lung cancer risk [95% confidence interval (CI) = 1.14−1.33, *P* = 9.08 × 10^−8^]. The regional association plot generated by using LocusZoom^22^ for rs114020893 ± 500 KB in the additive genetic model (Fig. 2) indicated that there were five other SNPs that showed moderate linkage disequilibrium (LD) with rs114020893.

**Figure 3:**
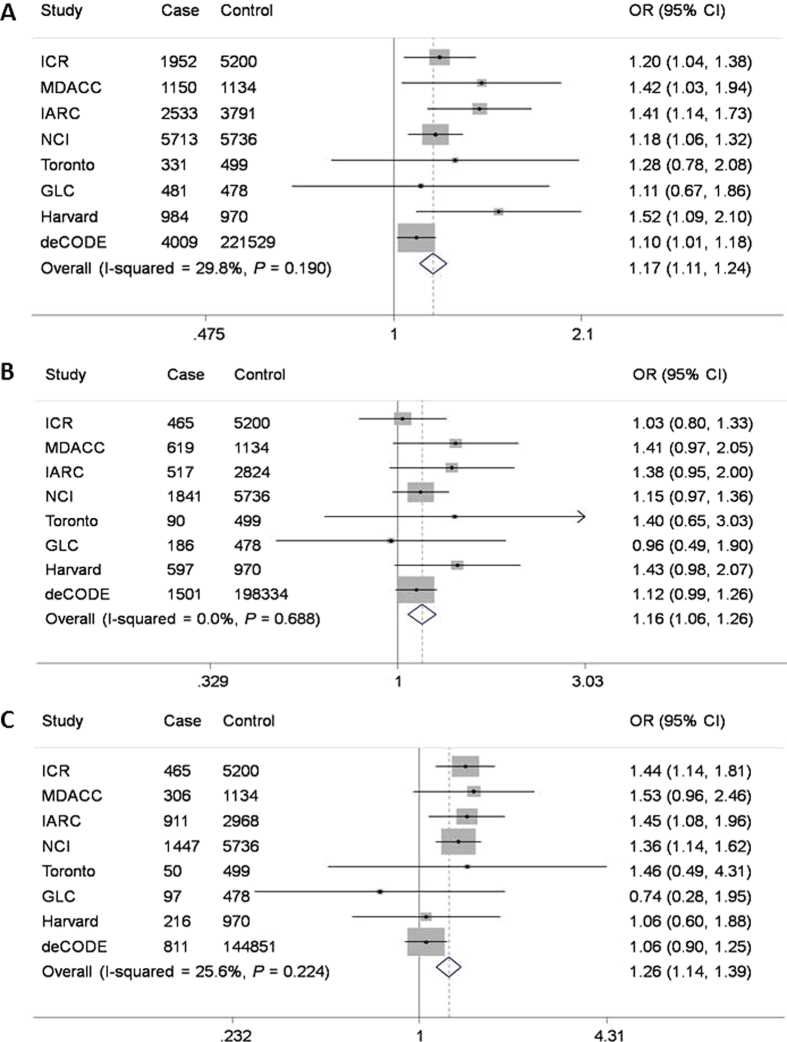
Forest plot of the C allele effect of rs114020893 in all cases (Panel A), adenocarcinoma (Panel B) and squamous cell carcinoma (Panel C) from the eight GWASs [the Institute of Cancer Research (ICR) GWAS, the MD Anderson Cancer Center (MDACC) GWAS, the International Agency for Research on Cancer (IARC) GWAS, the National Cancer Institute (NCI) GWAS, the Samuel Lunenfeld Research Institute study (Toronto) GWAS, German Lung Cancer Study (GLC), Harvard lung cancer study (Harvard) and Icelandic Lung Cancer Study (deCODE)].

We validated this initial finding by the data for the *NEXN-AS1* SNP rs114020893 from two additional independent lung cancer GWASs of Harvard University (984 cases and 970 controls) and deCODE (4,009 cases and 221,529 controls). As shown in Table 1, the rs114020893 SNP from both the Harvard and deCODE datasets was also significantly associated with risk of lung cancer (OR = 1.52, 95%CI = 1.10–2.11, *P* = 0.012 for Harvard and OR = 1.10, 95% CI = 1.01–1.18, *P* = 0.023 for deCODE), which were consistent with those derived from the six TRICL GWAS datasets. After pooling the data from all eight GWAS datasets, the rs114020893C allele was associated with a 1.17-fold (95% CI = 1.11–1.24) increased lung cancer risk, with a *P* value of 8.31 × 10^−9^, which remained statistically significant even after a conservative Bonferroni correction for 675,953 tests (Bonferroni-corrected significance cut-off: 7.24 × 10^−8^ from 0.05/690,564). Further subgroup analyses by tumor histology (Fig. 3) indicated that there was no difference in the association of rs114020893 with lung cancer risk between adenocarcinoma (OR = 1.16, 95% CI = 1.06–1.26) and squamous cell carcinoma (OR = 1.26, 95% CI = 1.14–1.39). The reason for this SNP not to be previously reported is because it is an imputed SNP in all the eight GWAS datasets, which suggests that other untyped SNPs could also have been missed by the published GWASs.

Considering the fact that rs114020893 is located within the lncRNA of *NEXN-AS1*, it is biologically plausible that rs114020893 may influence the function of *NEXN-AS1* by affecting its folding structure. Using the online tool RNAfold (http://rna.tbi.univie.ac.at/cgi-bin/RNAfold.cgi), it was predicted that rs114020893 could cause a change in the local lncRNA structure of *NEXN-AS1* as shown in Supplementary Figure 2, in which the SNP rs114020893 changes the folding structure of the *NEXN-AS1*.To determine in which tissues RNA transcripts (including *NEXN-AS1*) that include the SNP rs114020893 may be expressed, we utilized data from the ENCODE project, which systematically maps functional elements in the genome across a range of cell types^23^. Specifically, we scanned each RNA-seq experiment available at the ENCODE portal (http://www.encodeproject.org/) for which a GTF (Gene Transfer Format) file indicates that the transcribed regions in the human genome (build 37) were available as of June 23, 2015. From 1,011 such experiments, 156 had evidence for a gene in which rs114020893 is part of the expressed RNA. When we sorted these samples by gene abundance (either RPKM or FPKM), we observed that the highest levels of expression for genes that include this SNP were from two experiments on lung fibroblast tissue (experiments ENCSR000COO and ENCSR000CPM). This suggests that rs114020893 may alter a noncoding RNA that is expressed in lung fibroblasts, opening the door to testable mechanistic hypotheses.

## Discussion

Several loci including 5p15.33, 6p21.33 and 15q25.1 have been found to be associated with lung cancer risk in previously published GWASs^5^,^6^,^9^,^24^. However, these findings could only explain a small fraction of the risk of lung cancer. In the present study with an initial meta-analysis of six previously published GWAS datasets of the TRICL consortium, we systematically evaluated the associations of genetic variants within all lncRNAs that had been reported to date^17^, and we further validated the most promising association in two additional independent GWAS datasets of Harvard and deCODE lung cancer studies. As a result, we found that a novel SNP rs114020893 in the lncRNA gene *NEXN-AS1* located at 1p31.1 was significantly associated with lung cancer risk. This novel SNP was predicted to change the lncRNA structure. These findings suggest that genetic variation in the lncRNA regions may contribute to lung cancer etiology.

LncRNAs are often tissue-specific mRNA-like transcripts lacking significant open reading frames^16^. In various human tissues, lncRNAs are associated with development of diseases in a stage-specific manner, and functional lncRNAs may play a role in the development of cancer^25^. Emerging evidence suggests that differences in lncRNA expression levels are associated with the development of various types of cancer. For example, one notable lncRNA was discovered in the screening of genes associated with lung adenocarcinoma and was named metastasis-associated in lung adenocarcinoma transcript 1 (*MALAT1*)^26^. Additional studies found that *MALAT1* could regulate gene expression, especially for those that are involved in lung cancer cell migration, metastasis, and colony formation^27^. Furthermore, over expression of lncRNA *HOTAIR* in NSCLC tumors was found to be associated with advanced stages and shorter disease-free survival, and forced expression of *HOTAIR* induced cell migration and anchorage-independent-cell growth *in vitro*^28^. Expression changes in three additional lncRNAs have also been linked to lung cancer risk, including smoke and cancer-associated lncRNA-1 (*SCAL1*), GAS6-antisense 1, and maternally expressed gene 3 (*MEG3*)^29^,^30^,^31^. These lines of evidence suggest a crucial role of lncRNAs in lung carcinogenesis.

Several GWASs have been conducted to identify genetic susceptibility loci for lung cancer^5^,^6^. Subsequent bioinformatics analysis has also revealed several lncRNAs mapped to cancer-related genetic susceptibility loci^32^. In the present study, we found that lung cancer risk-related loci (6p21 and 15q25) were also enriched in lncRNAs, such as *RP11-650L12.2*, *HCP5*, *XXbac-BPG27H4.8*, and *HCG17*. In this GWAS re-analysis study, we aimed to demonstrate the possibility that genetic variants in lncRNA regions might be associated with lung cancer development. This method of using non-coding regions should be complementary to the protein-coding-related approaches, such as the gene-based and pathway-based analyses^33^,^34^.

By systematically analyzing SNPs in lncRNAs, we identified a novel lung cancer risk locus (1p31.1), which harbors a potentially functional SNP rs114020893 in lncRNA *NEXN-AS1*. This region has also been implicated in GWASs of two diseases: Crohn’s disease^35^ and class II obesity^31^. To date, however, the potential biological mechanism underlying these findings remains unknown. Because rs114020893 is located on the exon of the *NEXN-AS1* gene, the *in-silico* analyses predicted the influence of the T/C alleles of rs114020893 on the secondary structure of *NEXN-AS1*. As a result, the secondary structure was remarkably changed with the rs114020893 T > C change, indicating that this SNP may be involved in lung cancer development through alteration of the *NEXN-AS1* structure and stability, resulting in the functional alteration of its interacting partners. Further studies on additional SNPs and the biological mechanisms of the *NEXN-AS1* gene are warranted.

To our knowledge, this is the first study investigating the role of genetic variants of lncRNAs in lung cancer susceptibility at the whole-genome level, although the roles of lncRNAs in carcinogenesis of some other cancers have been reported. Our observation may provide a novel insight into the roles of lncRNAs in lung cancer etiology. The large sample size, ensuring sufficient statistical power to detect small effect size, is the other strength of the present study. However, there are some limitations that need to be mentioned. Firstly, our list of lncRNAs may not be comprehensive, because we were limited by those that have already been identified to date and those included in the GENCODE database (15,531 autosomal lncRNAs). Secondly, the classification of lncRNAs has not been functionally characterized, and thus little is known about the biological meanings of lncRNAs, which makes explanation of our results difficult. Thirdly, the identified novel SNP was imputed, and thus its real effect size may need to be validated in actual genotyping data in the future. Finally, it is still unclear how such a SNP modifies the formation or effects of lncRNAs. This SNP is also located at the 345 bp upstream of the *NEXN* gene, but its functional relevance is still not clear. It is possible that the SNP may function through the lncRNA *NEXN-AS1* or play a regulatory role of the encoding gene *NEXN*. Further functional analysis of the variant is warranted.

In conclusion, based on the results from a large meta-analysis of eight published GWASs of European descent, we have identified a novel SNP rs114020893 T > C, located in the lncRNA *NEXN-AS1* gene that is significantly associated with an increased risk of lung cancer. To confirm the biological significance of our finding, further functional analysis of the variant is warranted.

## Materials and Methods

### Study populations

The meta-analysis first used the combined genotyping and imputation dataset of six previously published GWASs of lung cancer with 12,160 lung cancer cases and 16,838 controls of European ancestry from the TRICL consortium and International Lung Cancer Consortium^8^,^21^,^24^. As shown in Supplementary Table 2, these six published studies included the Institute of Cancer Research (ICR) GWAS, the MD Anderson Cancer Center (MDACC) GWAS, the International Agency for Research on Cancer (IARC) GWAS, the National Cancer Institute (NCI) GWAS, the Samuel Lunenfeld Research Institute study (Toronto) GWAS, and the German Lung Cancer Study (GLC)^21^. Additional two datasets of independent GWASs of Caucasian populations was used: the Harvard Lung Cancer Study (Harvard) which includes 984 cases and 970 controls and the Icelandic Lung Cancer Study (deCODE), which, in addition to using data from chip typed individuals, also allows inclusion of individuals that have not been chip typed, but for which genotype probabilities are imputed using methods of familial imputation. The effective sample size of deCODE’s dataset is 6,612cases and 6,612 controls^8^ (Supplementary Table 2). A written informed consent was obtained from each participant of these GWASs, and the present study followed the study protocols approved by the institutional review board for each of the participating institutions.

### Selection of lncRNA genes and SNPs

We selected 15,900 lncRNAs from the publically available database GENCODE Release22 (GRCh38; released in March, 2015)^16^,^36^. All genotyping was performed by one of Illumina HumanHap 317, 317 + 240S, 370Duo, 550, 610 or 1 M arrays^8^,^21^. The genotyping data were also used for imputation from all scans for over 10 million SNPs from the 1000 Genomes Project (phase I integrated release 3, March 2012) as the reference by using IMPUTE2 v2.1.1, MaCH v1.0 or minimac (version 2012.10.3) software. The quality control process has been detailed in previous reports^8^,^21^. As a result, the final dataset included 15,531 lncRNA genes located on autosomes, and 690,564 genotyped or imputed common [minor allele frequency (MAF) > 0.05] SNPs within these lncRNA genes were used for association analyses. SNPs with a *P* value < 1 × 10^−7^ in the TRICL datasets were further tested in the Harvard and deCODE datasets, excluding those SNPs located in the regions that had been reported to contribute to lung cancer risk in previously published studies^5^,^6^,^9^,^24^. The detailed work-flow is shown in Supplementary Figure 3.

### Functional validation

We used the online tool RNAfold (http://rna.tbi.univie.ac.at/cgi-bin/RNAfold.cgi) to predict the effects of identified SNPs on the change of lncRNA. We also scanned the data from RNA-seq experiments available at the Encyclopedia of DNA Elements (ENCODE) portal (http://www.encodeproject.org/) to determine in which tissues RNA transcripts (including NEXN-AS1) that include the identified SNPs may be expressed^23^.

### Statistical methods

The statistical methods have been detailed in a previous publication^8^. Briefly, the association between each SNP and lung cancer risk was assessed by an additive genetic model of the minor allele, using R (v2.6), Stata v1.0 (Stata College, Texas, US), SAS software (version 9.3; SAS Institute, Cary, NC, USA) and Plink (v1.06) software. Specifically, poorly imputed SNPs defined by an information score <0.40 with IMPUTE2 or an r-square <0.30 with MaCH were excluded from the analyses. The *I*^2^ statistic to quantify the proportion of the total variation due to the heterogeneity and the Chi-square-based Cohran’s Q statistic to test for heterogeneity were calculated^37^. Fixed-effects models were applied when there was no heterogeneity among studies (*P* > 0.100 and *I*^2^ < 50%); otherwise, random-effects models were applied. The full sequences of *NEXN-AS1* (NCBI Reference Sequence: NR_103535.1) containing T or C alleles in rs114020893 were used to predict the folding structures of *NEXN-AS1* in RNAfold (http://rna.tbi.univie.ac.at/cgi-bin/RNAfold.cgi)^38^. ORs and their 95% CI were used to estimate cancer risk associated with the variant allele or genotypes.

## Additional Information

**How to cite this article**: Yuan, H. *et al*. A Novel Genetic Variant in Long Non-coding RNA Gene *NEXN-AS1* is Associated with Risk of Lung Cancer. *Sci. Rep*. **6**, 34234; doi: 10.1038/srep34234 (2016).

## Supplementary Material

Supplementary Information

## Figures and Tables

**Table 1:** Summary of the association results of rs114020893 in the eight lung cancer GWASs.

Study population	Sample size		rs114020893 (C)	
	Cases	Controls	OR (95%CI)	*P*
**TRICL combined**^1^	**12160**	**16838**	**1.23 (1.14−1.33)**	**9.08E-08**
ICR^2^	1952	5200	1.20 (1.04−1.38)	1.13E-02
MDACC^3^	1150	1134	1.42 (1.03−1.94)	3.00E-02
IARC^4^	2533	3791	1.41 (1.14−1.73)	1.16E-03
NCI^5^	5713	5736	1.18 (1.06−1.32)	2.80E-03
Toronto^6^	331	499	1.28 (0.78−2.08)	3.28E-01
GLC^7^	481	478	1.11 (0.67−1.86)	6.77E-01
**Replicationcombined**^1^	**4993**	**222499**	**1.11 (1.03−1.20)**	**5.17E-03**
Harvard^8^	984	970	1.52 (1.10−2.11)	1.23E-02
deCODE^9^	4009	221529	1.10 (1.01−1.18)	2.29E-02
**All combined**^1^	**17153**	**239337**	**1.17 (1.11−1.24)**	**8.31E-09**

^1^The combined OR and *P* value were estimated using a fixed-effects model;

^2^ICR: the Institute of Cancer Research Genome-wide Association Study, UK;

^3^MDACC: The University of Texas MD Anderson Cancer Center Genome-wide Association Study, US;

^4^IARC: the International Agency for Research on Cancer Genome-wide Association Study, France;

^5^NCI: the National Cancer Institute Genome-wide Association Study, US;

^6^Toronto: the Samuel Lunenfeld Research Institute Genome-wide Association Study, Toronto, Canada;

^7^GLC: German Lung Cancer Study, Germany;

^8^Harvard: Harvard Lung Cancer Study, US;

^9^deCODE: Icelandic Lung Cancer Study, Iceland.
