# Supplementary material for: A Novel Genetic Variant in Long Non-coding RNA Gene NEXN-AS1 is Associated with Risk of Lung Cancer
Source: Sci Rep. 2016 Oct 7;6:34234. doi: 10.1038/srep34234 (PMC5054367; doi:10.1038/srep34234)
Supplement: Supplementary Information [file srep34234-s1.doc]

**A Novel Genetic Variant in Long Non-coding RNA Gene *NEXN-AS1* is Associated with Risk of Lung Cancer**

Hua Yuan1,2,3† , Hongliang Liu1,2† , Zhensheng Liu1,2, Dakai Zhu4 , Christopher I. Amos4 , Shenying Fang5 , Jeffrey E. Lee5 and Qingyi Wei1,2 1 Duke Cancer Institute, Duke University Medical Center, Durham, NC 2 Department of Medicine, Duke University School of Medicine, Durham, NC 3 Department of Oral and Maxillofacial Surgery, Affiliated Hospital of Stomatology, Nanjing Medical University, Nanjing, China 4 Community and Family Medicine, Geisel School of Medicine, Dartmouth College, Hanover, NH 5 Department of Surgical Oncology, The University of Texas M. D. Anderson Cancer Center, Houston, TXTheSaban Research Institute of Children’s Hospital Los Angeles, Los Angeles, CA

2

Division of Hematology, Oncology, and Blood & Marrow Transplantation, University of Southern California Keck School of Medicine, Los Angeles, CA

3

Department of Pediatrics, University of Southern California Keck School of Medicine, Los Angeles, CA

4

Department of Orthopedic Surgery, University of Southern California, Los Angeles, CA

5

Department of Biochemistry & Molecular Biology, University of Southern California, Los Angeles, CA

6

Norris Comprehensive Cancer Center Bioinformatics Core and Division of Hematology, University of Southern California Keck School of Medicine, Los

Angeles, CA

Hua Yuan^1,2,3^, Hongliang Liu^2,3^ , Zhensheng Liu^2,3^, Kouros Owzar^3,4^, Younghun Han^5^, Li Su^6,7^, Yongyue Wei^6,7^, Rayjean J. Hung^8^, John McLaughlin^9^, Yonathan Brhane^8^, Paul Brennan^10^, Heike Bickeboeller^11^, Albert Rosenberger^11^, Richard S. Houlston^12^, Neil Caporaso^13^, Maria Teresa Landi^13^, Joachim Heinrich^14,15^, Angela Risch^16,17,18^, David C. Christiani^6,7^, Zeynep H. Gümüş^19^, Robert J. Klein^19^, Christopher I. Amos^5^, and Qingyi Wei^2,3^

^1^ Collaborative Innovation Center For Cancer Personalized Medicine, Nanjing Medical University; Department of Oral and Maxillofacial Surgery, Affiliated Hospital of Stomatology, Nanjing Medical University, Nanjing, China

^2^ Department of Medicine, Duke University School of Medicine, Durham, NC

^3^ Duke Cancer Institute, Duke University Medical Center, Durham, NC

^4^ Department of Biostatistics and Bioinformatics, Duke University Medical Center, Durham, NC

^5^ Community and Family Medicine, Geisel School of Medicine, Dartmouth College, Hanover, NH

^6^ Massachusetts General Hospital, Boston, Massachusetts.

^7^ Department of Environmental Health, Harvard School of Public Health, Boston, Massachusetts.

^8^ Lunenfeld-Tanenbaum Research Institute of Mount Sinai Hospital, Toronto, Ontario, Canada.

^9^ Public Health Ontario, Toronto, Ontario, Canada

^10^ Genetic Epidemiology Group, International Agency for Research on Cancer (IARC), Lyon, France

^11^ Department of Genetic Epidemiology, University Medical Center, Georg-August-University Göttingen, Göttingen, Germany.

^12^ Division of Genetics and Epidemiology, The Institute of Cancer Research, London, United Kingdom

^13^ Genetic Epidemiology Branch, Division of Cancer Epidemiology and Genetics, National Cancer Institute, National Institutes of Health, Bethesda, MD, USA

^14^ Helmholtz Centre Munich, German Research Centre for Environmental Health, Institute of Epidemiology I, Neuherberg, Germany

^15^ Institute and Outpatient Clinic for Occupational, Social and Environmental Medicine, University Hospital Munich, Ludwig Maximilian University Munich, Munich, Germany

^16^ Department of Molecular Biology, University of Salzburg, Salzburg, Austria

^17^ Department of Epigenomics and Cancer Risk Factors, DKFZ - German Cancer Research Center, Heidelberg, Germany

^18^ Translational Lung Research Center Heidelberg (TLRC-H), Member of the German Center for Lung Research (DZL), Heidelberg, Germany.

^19^ Department of Genetics and Genomic Sciences, Icahn Institute for Genomics and Multiscale Biology, Icahn School of Medicine at Mount Sinai, New York, USA

Correspondence to: Qingyi Wei, M.D., Ph.D., Duke Cancer Institute, Duke University Medical Center, 905 S. Lasalle Street, Durham, NC 27710, USA, Tel.: 1-(919) 660-0562, E-mail: qingyi.wei@duke.edu

**Supplementary Table 1.** Associations between SNPs in the lncRNAs and lung cancer risk with a *P* value<1 × 10^-7^ in the six GWAS datasets

| **SNP** | **Chr** | **Position^a^** | **lncRNA** | **Allele^b^** | **EAF^c^** | ***P*_hete_^d^** | ***I*^2^** | **Effects^e^** | **OR (95%CI)^f^** | ***P*^f^** |
| --- | --- | --- | --- | --- | --- | --- | --- | --- | --- | --- |
| rs16969968 | 15 | 78882925 | *RP11-650L12.2* | G/A | 0.369 | 0.896 | 0.000 | ++++++ | 1.35(1.30-1.40) | 4.94E-61 |
| rs17484524 | 15 | 78772676 | *RP11-650L12.1* | A/G | 0.359 | 0.182 | 33.959 | ++++++ | 1.30(1.24-1.37) | 1.35E-43 |
| rs17408276 | 15 | 78881618 | *RP11-650L12.2* | T/C | 0.360 | 0.551 | 0.000 | ------ | 0.88(0.85-0.92) | 1.77E-11 |
| [rs3094604](http://www.ncbi.nlm.nih.gov/projects/SNP/snp_ref.cgi?rs=3094604) | 6 | 31434111 | *HCP5* | A/G | 0.183 | 0.695 | 0.000 | ++++++ | 1.17(1.11-1.22) | 5.89E-11 |
| [rs3130907](http://www.ncbi.nlm.nih.gov/projects/SNP/snp_ref.cgi?rs=3130907) | 6 | 31431813 | *HCP5* | A/G | 0.121 | 0.738 | 0.000 | ++++++ | 1.19(1.13-1.26) | 3.96E-10 |
| [rs3132089](http://www.ncbi.nlm.nih.gov/projects/SNP/snp_ref.cgi?rs=3132089) | 6 | 31430010 | *HCP5* | G/A | 0.121 | 0.736 | 0.000 | ++++++ | 1.19(1.13-1.26) | 4.00E-10 |
| [rs3099839](http://www.ncbi.nlm.nih.gov/projects/SNP/snp_ref.cgi?rs=3099839) | 6 | 31430065 | *HCP5* | C/T | 0.121 | 0.737 | 0.000 | ++++++ | 1.19(1.13-1.26) | 4.00E-10 |
| [rs3094605](http://www.ncbi.nlm.nih.gov/projects/SNP/snp_ref.cgi?rs=3094605) | 6 | 31430694 | *HCP5* | G/C | 0.121 | 0.738 | 0.000 | ++++++ | 1.19(1.13-1.26) | 4.01E-10 |
| [rs3130477](http://www.ncbi.nlm.nih.gov/projects/SNP/snp_ref.cgi?rs=3130477) | 6 | 31428920 | *HCP5* | T/C | 0.121 | 0.734 | 0.000 | ++++++ | 1.19(1.13-1.26) | 4.10E-10 |
| [rs3132090](http://www.ncbi.nlm.nih.gov/projects/SNP/snp_ref.cgi?rs=3132090) | 6 | 31430752 | *HCP5* | G/A | 0.120 | 0.735 | 0.000 | ++++++ | 1.19(1.13-1.26) | 4.15E-10 |
| [rs3128986](http://www.ncbi.nlm.nih.gov/projects/SNP/snp_ref.cgi?rs=3128986) | 6 | 31433693 | *HCP5* | T/C | 0.121 | 0.751 | 0.000 | ++++++ | 1.19(1.13-1.26) | 4.29E-10 |
| [rs2517572](http://www.ncbi.nlm.nih.gov/projects/SNP/snp_ref.cgi?rs=2517572) | 6 | 30842629 | *DDR1-AS1* | C/T | 0.114 | 0.462 | 0.000 | ++++++ | 1.19(1.12-1.25) | 4.46E-10 |
| [rs3131619](http://www.ncbi.nlm.nih.gov/projects/SNP/snp_ref.cgi?rs=3131619) | 6 | 31434331 | *HCP5* | A/T | 0.121 | 0.768 | 0.000 | ++++++ | 1.19(1.13-1.26) | 4.98E-10 |
| [rs3094013](http://www.ncbi.nlm.nih.gov/projects/SNP/snp_ref.cgi?rs=3094013) | 6 | 31434366 | *HCP5* | G/A | 0.121 | 0.769 | 0.000 | ++++++ | 1.19(1.13-1.26) | 5.01E-10 |
| [rs2535340](http://www.ncbi.nlm.nih.gov/projects/SNP/snp_ref.cgi?rs=2535340) | 6 | 30838497 | *DDR1-AS1* | T/C | 0.114 | 0.476 | 0.000 | ++++++ | 1.19(1.12-1.25) | 6.01E-10 |
| [rs2535334](http://www.ncbi.nlm.nih.gov/projects/SNP/snp_ref.cgi?rs=2535334) | 6 | 30812996 | *XXbac-BPG27H4.8* | G/A | 0.114 | 0.471 | 0.000 | ++++++ | 1.19(1.12-1.25) | 6.04E-10 |
| [rs2535332](http://www.ncbi.nlm.nih.gov/projects/SNP/snp_ref.cgi?rs=2535332) | 6 | 30813249 | *XXbac-BPG27H4.8* | C/T | 0.114 | 0.471 | 0.000 | ++++++ | 1.19(1.12-1.25) | 6.04E-10 |
| [rs2517578](http://www.ncbi.nlm.nih.gov/projects/SNP/snp_ref.cgi?rs=2517578) | 6 | 30811265 | *XXbac-BPG27H4.8* | G/C | 0.114 | 0.471 | 0.000 | ++++++ | 1.19(1.12-1.25) | 6.11E-10 |
| rs62010332 | 15 | 78918762 | *RP11-335K5.2* | G/C | 0.340 | 0.349 | 10.430 | -----+ | 0.89(0.86-0.92) | 8.22E-10 |
| [rs3130380](http://www.ncbi.nlm.nih.gov/projects/SNP/snp_ref.cgi?rs=3130380) | 6 | 30279130 | *HCG17* | G/A | 0.101 | 0.471 | 0.000 | ++++++ | 1.19(1.13-1.26) | 1.07E-09 |
| [rs3129830](http://www.ncbi.nlm.nih.gov/projects/SNP/snp_ref.cgi?rs=3129830) | 6 | 30248711 | *HCG17* | G/A | 0.102 | 0.495 | 0.000 | ++++++ | 1.19(1.13-1.26) | 1.08E-09 |
| [rs3094069](http://www.ncbi.nlm.nih.gov/projects/SNP/snp_ref.cgi?rs=3094069) | 6 | 30251391 | *HCG17* | C/T | 0.102 | 0.495 | 0.000 | ++++++ | 1.19(1.13-1.26) | 1.09E-09 |
| [rs3115631](http://www.ncbi.nlm.nih.gov/projects/SNP/snp_ref.cgi?rs=3115631) | 6 | 29986324 | *ZNRD1-AS1* | T/A | 0.098 | 0.456 | 0.000 | ++++++ | 1.20(1.13-1.27) | 1.14E-09 |
| [rs3131618](http://www.ncbi.nlm.nih.gov/projects/SNP/snp_ref.cgi?rs=3131618) | 6 | 31434621 | *HCP5* | A/G | 0.117 | 0.729 | 0.000 | ++++++ | 1.19(1.13-1.26) | 1.18E-09 |
| [rs3094078](http://www.ncbi.nlm.nih.gov/projects/SNP/snp_ref.cgi?rs=3094078) | 6 | 30224970 | *HCG17* | T/A | 0.102 | 0.454 | 0.000 | ++++++ | 1.19(1.13-1.26) | 1.50E-09 |
| [rs3094146](http://www.ncbi.nlm.nih.gov/projects/SNP/snp_ref.cgi?rs=3094146) | 6 | 29970960 | *ZNRD1-AS1* | G/C | 0.099 | 0.411 | 0.869 | ++++++ | 1.19(1.12-1.26) | 2.55E-09 |
| rs35031105 | 15 | 78772806 | *RP11-650L12.1* | C/T | 0.212 | 0.077 | 49.741 | ------ | 0.81(0.76-0.87) | 2.75E-09 |
| [rs3094066](http://www.ncbi.nlm.nih.gov/projects/SNP/snp_ref.cgi?rs=3094066) | 6 | 30278745 | *HCG17* | C/T | 0.103 | 0.495 | 0.000 | ++++++ | 1.18(1.12-1.25) | 4.14E-09 |
| [rs3094075](http://www.ncbi.nlm.nih.gov/projects/SNP/snp_ref.cgi?rs=3094075) | 6 | 30229267 | *HCG17* | G/C | 0.103 | 0.507 | 0.000 | ++++++ | 1.18(1.12-1.25) | 4.26E-09 |
| [rs3130399](http://www.ncbi.nlm.nih.gov/projects/SNP/snp_ref.cgi?rs=3130399) | 6 | 30230768 | *HCG17* | A/T | 0.103 | 0.507 | 0.000 | ++++++ | 1.18(1.12-1.25) | 4.33E-09 |
| [rs9267123](http://www.ncbi.nlm.nih.gov/projects/SNP/snp_ref.cgi?rs=9267123) | 6 | 31427395 | *HCP5* | G/C | 0.135 | 0.830 | 0.000 | ++++++ | 1.17(1.11-1.23) | 4.91E-09 |
| [rs3132685](http://www.ncbi.nlm.nih.gov/projects/SNP/snp_ref.cgi?rs=3132685) | 6 | 29945949 | *HCG9* | G/A | 0.109 | 0.388 | 4.509 | ++++++ | 1.18(1.12-1.25) | 4.91E-09 |
| [rs3128987](http://www.ncbi.nlm.nih.gov/projects/SNP/snp_ref.cgi?rs=3128987) | 6 | 31434198 | *HCP5* | T/C | 0.191 | 0.830 | 0.000 | ++++++ | 1.15(1.09-1.20) | 5.12E-09 |
| [rs3131620](http://www.ncbi.nlm.nih.gov/projects/SNP/snp_ref.cgi?rs=3131620) | 6 | 31433831 | *HCP5* | A/G | 0.191 | 0.827 | 0.000 | ++++++ | 1.15(1.09-1.20) | 5.53E-09 |
| [rs3094014](http://www.ncbi.nlm.nih.gov/projects/SNP/snp_ref.cgi?rs=3094014) | 6 | 31433558 | *HCP5* | G/A | 0.191 | 0.824 | 0.000 | ++++++ | 1.15(1.09-1.20) | 5.90E-09 |
| [rs3094628](http://www.ncbi.nlm.nih.gov/projects/SNP/snp_ref.cgi?rs=3094628) | 6 | 30285312 | *HCG17* | G/C | 0.104 | 0.442 | 0.000 | ++++++ | 1.19(1.12-1.26) | 6.01E-09 |
| [rs2844777](http://www.ncbi.nlm.nih.gov/projects/SNP/snp_ref.cgi?rs=2844777) | 6 | 30206014 | *HCG17* | G/A | 0.103 | 0.499 | 0.000 | ++++++ | 1.18(1.12-1.25) | 6.20E-09 |
| [rs2844781](http://www.ncbi.nlm.nih.gov/projects/SNP/snp_ref.cgi?rs=2844781) | 6 | 30204247 | *HCG17* | A/T | 0.103 | 0.523 | 0.000 | ++++++ | 1.18(1.12-1.25) | 6.56E-09 |
| [rs3131617](http://www.ncbi.nlm.nih.gov/projects/SNP/snp_ref.cgi?rs=3131617) | 6 | 31436738 | *HCP5* | A/T | 0.121 | 0.821 | 0.000 | ++++++ | 1.17(1.11-1.24) | 7.43E-09 |
| [rs1264373](http://www.ncbi.nlm.nih.gov/projects/SNP/snp_ref.cgi?rs=1264373) | 6 | 30769273 | *LINC00243* | G/A | 0.112 | 0.605 | 0.000 | ++++++ | 1.17(1.11-1.24) | 8.33E-09 |
| rs2524266 | 6 | 30780568 | *LINC00243* | C/T | 0.112 | 0.564 | 0.000 | ++++++ | 1.17(1.11-1.24) | 1.01E-08 |
| [rs1264357](http://www.ncbi.nlm.nih.gov/projects/SNP/snp_ref.cgi?rs=1264357) | 6 | 30783579 | *LINC00243* | G/A | 0.112 | 0.568 | 0.000 | ++++++ | 1.17(1.11-1.24) | 1.10E-08 |
| [rs2023473](http://www.ncbi.nlm.nih.gov/projects/SNP/snp_ref.cgi?rs=2023473) | 6 | 30075613 | *TRIM31-AS1* | T/C | 0.102 | 0.439 | 0.000 | ++++++ | 1.18(1.11-1.25) | 1.13E-08 |
| [rs3131788](http://www.ncbi.nlm.nih.gov/projects/SNP/snp_ref.cgi?rs=3131788) | 6 | 31024796 | *HCG22* | G/A | 0.115 | 0.630 | 0.000 | ++++++ | 1.17(1.11-1.23) | 1.32E-08 |
| [rs3132600](http://www.ncbi.nlm.nih.gov/projects/SNP/snp_ref.cgi?rs=3132600) | 6 | 30746367 | *HCG20* | C/T | 0.111 | 0.673 | 0.000 | ++++++ | 1.17(1.11-1.24) | 1.49E-08 |
| [rs1264349](http://www.ncbi.nlm.nih.gov/projects/SNP/snp_ref.cgi?rs=1264349) | 6 | 30796659 | *LINC00243* | A/G | 0.112 | 0.572 | 0.000 | ++++++ | 1.17(1.11-1.24) | 1.55E-08 |
| [rs1264351](http://www.ncbi.nlm.nih.gov/projects/SNP/snp_ref.cgi?rs=1264351) | 6 | 30792117 | *LINC00243* | G/C | 0.112 | 0.578 | 0.000 | ++++++ | 1.17(1.11-1.23) | 1.62E-08 |
| rs3130668 | 6 | 30743729 | *HCG20* | G/A | 0.101 | 0.672 | 0.000 | ++++++ | 1.19(1.12-1.26) | 1.68E-08 |
| [rs3132580](http://www.ncbi.nlm.nih.gov/projects/SNP/snp_ref.cgi?rs=3132580) | 6 | 30920124 | *HCG21* | G/A | 0.133 | 0.418 | 0.000 | ++++++ | 1.16(1.10-1.22) | 1.90E-08 |
| [rs3094086](http://www.ncbi.nlm.nih.gov/projects/SNP/snp_ref.cgi?rs=3094086) | 6 | 30919391 | *HCG21* | G/A | 0.135 | 0.318 | 14.938 | ++++++ | 1.15(1.10-1.21) | 2.48E-08 |
| [rs3129693](http://www.ncbi.nlm.nih.gov/projects/SNP/snp_ref.cgi?rs=3129693) | 6 | 30207929 | *HCG17* | A/T | 0.157 | 0.821 | 0.000 | ++++++ | 1.15(1.09-1.20) | 3.17E-08 |
| [rs3094228](http://www.ncbi.nlm.nih.gov/projects/SNP/snp_ref.cgi?rs=3094228) | 6 | 31429927 | *HCP5* | T/C | 0.216 | 0.513 | 0.000 | ++++++ | 1.13(1.08-1.18) | 3.27E-08 |
| [rs3099840](http://www.ncbi.nlm.nih.gov/projects/SNP/snp_ref.cgi?rs=3099840) | 6 | 31430721 | *HCP5* | A/G | 0.216 | 0.521 | 0.000 | ++++++ | 1.13(1.08-1.18) | 3.42E-08 |
| rs146423591 | 6 | 30218379 | *HCG17* | C/T | 0.115 | 0.446 | 0.000 | ++++++ | 1.17(1.10-1.23) | 4.07E-08 |
| [rs2517601](http://www.ncbi.nlm.nih.gov/projects/SNP/snp_ref.cgi?rs=2517601) | 6 | 30074072 | *TRIM31-AS1* | G/C | 0.106 | 0.365 | 8.078 | ++++++ | 1.17(1.11-1.24) | 4.61E-08 |
| [rs1264356](http://www.ncbi.nlm.nih.gov/projects/SNP/snp_ref.cgi?rs=1264356) | 6 | 30784068 | *LINC00243* | C/T | 0.123 | 0.568 | 0.000 | ++++++ | 1.16(1.10-1.22) | 6.17E-08 |
| [rs3094012](http://www.ncbi.nlm.nih.gov/projects/SNP/snp_ref.cgi?rs=3094012) | 6 | 31434520 | *HCP5* | G/C | 0.129 | 0.850 | 0.000 | ++++++ | 1.16(1.10-1.22) | 8.68E-08 |
| rs114020893 | 1 | 78353605 | *NEXN-AS1* | T/C | 0.085 | 0.672 | 0.000 | ++++++ | 1.23 (1.14-1.33) | 9.08E-08 |
| [rs886424](http://www.ncbi.nlm.nih.gov/projects/SNP/snp_ref.cgi?rs=886424) | 6 | 30782002 | *LINC00243* | C/T | 0.119 | 0.565 | 0.000 | +++++- | 1.15(1.10-1.22) | 9.60E-08 |
| ^a^Based on NCBI build 37 of the human genome.  ^b^Reference allele/effect allele.  ^c^Effect allele frequency.  *^d^P* value for Cochran's heterogeneity test.  ^e^Effects by study: ICR, MDACC, IARC, NCI, SLRI, and HGF Germany, respectively. + represents OR > 1.00, and - represents OR < 1.00.  ^f^Meta-analysis additive model *P*-value based on six lung cancer GWASs. | | | | | | | | | | |

**Supplementary Table 2**. Characteristics of the study populations by tumor type included in the participating GWASs

| **Variable** | **ICR^1^** | |  | **MDACC^2^** | |  | **IARC^3^** | |  | **NCI^4^** | |  | **Toronto 5** | |  | **GLC Germany^6^** | |  | **Harvard^7^** | |  | **deCODE^8^** | |
| --- | --- | --- | --- | --- | --- | --- | --- | --- | --- | --- | --- | --- | --- | --- | --- | --- | --- | --- | --- | --- | --- | --- | --- |
|  | **Case** | **Control** |  | **Case** | **Control** |  | **Case** | **Control** |  | **Case** | **Control** |  | **Case** | **Control** |  | **Case** | **Control** |  | **Case** | **Control** |  | **Case** | **Control** |
| **Overall** | 1952 | 5200 |  | 1150 | 1134 |  | 2533 | 3791 |  | 5713 | 5736 |  | 331 | 499 |  | 481 | 478 |  | 984 | 970 |  | 4009 | 221529 |
| **AD** | 465 | 5200 |  | 619 | 1134 |  | 517 | 2824 |  | 1841 | 5736 |  | 90 | 499 |  | 186 | 478 |  | 597 | 970 |  | 1501 | 198334 |
| **SQ** | 611 | 5200 |  | 306 | 1134 |  | 911 | 2968 |  | 1447 | 5736 |  | 50 | 499 |  | 97 | 478 |  | 216 | 970 |  | 811 | 144851 |

AD: adenocarcinoma, SQ: squamous cell carcinoma.

^1^ ICR: the Institute of Cancer Research Genome-wide Association Study, UK;

^2^ MDACC: the MD Anderson Cancer Center Genome-wide Association Study, US;

^3^ IARC: the International Agency for Research on Cancer Genome-wide Association Study,France;

^4^ NCI: the National Cancer Institute Genome-wide Association Study, US;

^5^Toronto: the Samuel Lunenfeld Research Institute Genome-wide Association Study, Toronto, Canada;

^6^ GLC: German Lung Cancer Study, Germany;

^7^Harvard: Harvard Lung Cancer Study, US;

^8^deCODE: Icelandic Lung Cancer Study, Iceland.

**Supplementary Figure 1.** Forest plot of the C allele effect of rs114020893 in the six discovery datasets from TRICL consortium. The TRICL GWAS meta-analysis includes six GWASs: the Institute of Cancer Research (ICR) GWAS, the MD Anderson Cancer Center (MDACC) GWAS, the International Agency for Research on Cancer (IARC) GWAS, the National Cancer Institute (NCI) GWAS, the Samuel Lunenfeld Research Institute study (Toronto) GWAS and German Lung Cancer Study (GLC).

Overall (I-squared = 0.0%, *P* = 0.672)

IARC

Study

ICR

SLRI

MDACC

NCI

GLC

2533

Case

1952

331

1150

5713

481

3791

Control

5200

499

1134

5736

478

1.23 (1.14, 1.33)

1.41 (1.14, 1.73)

1.20 (1.04, 1.38)

1.28 (0.78, 2.08)

1.42 (1.03, 1.94)

1.18 (1.06, 1.32)

1.11 (0.67, 1.86)

OR (95% CI)

0

1

1.8


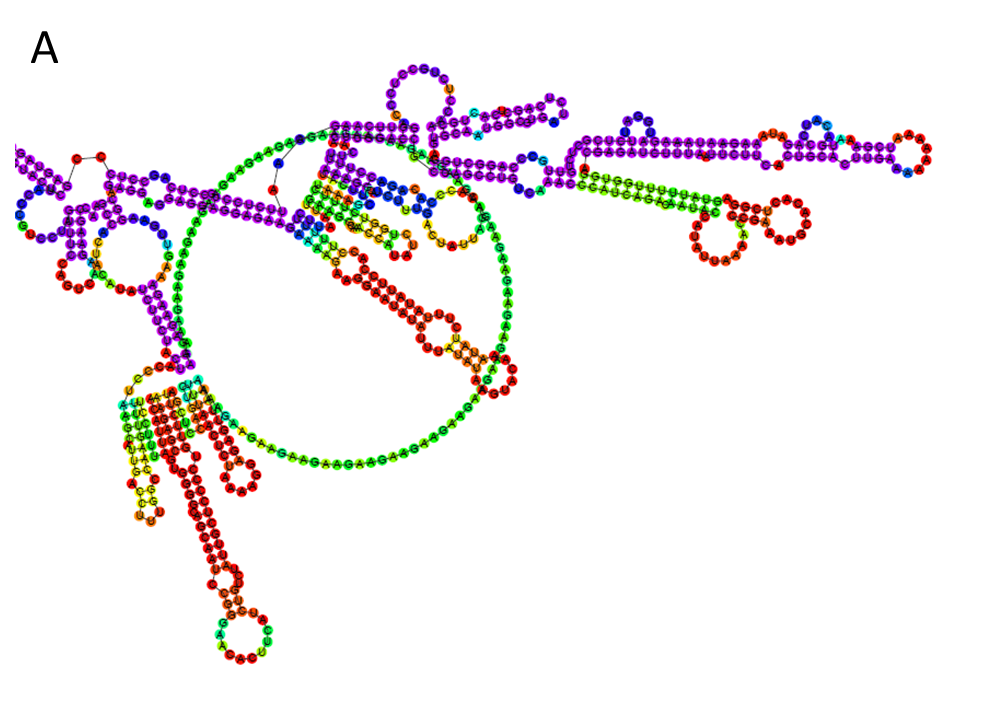


**
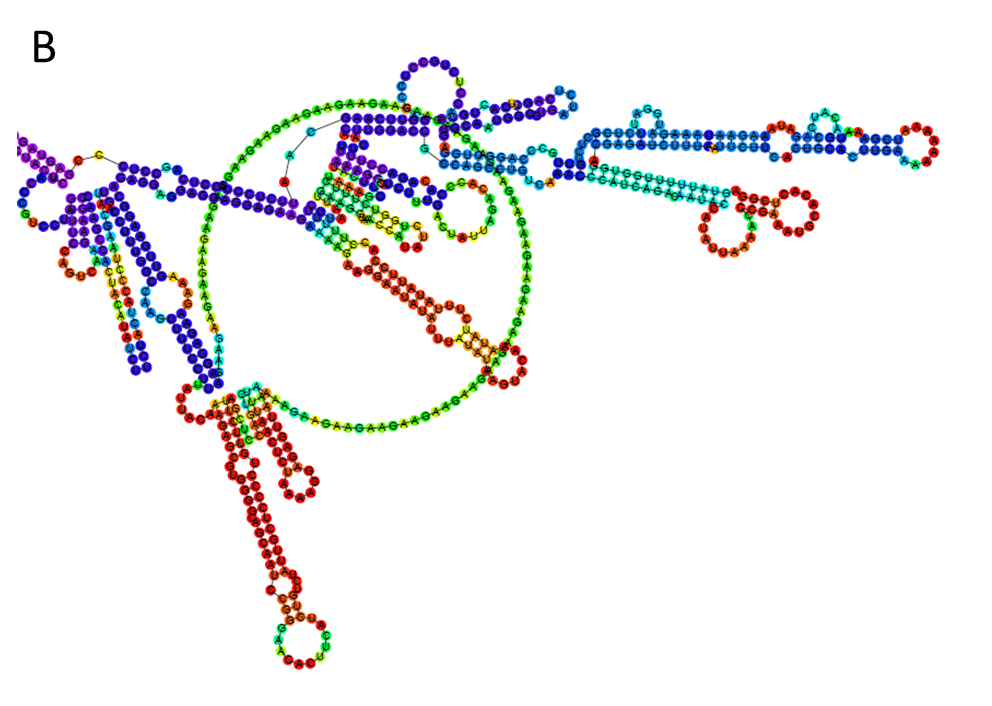
**

**Supplementary Figure 2**. *In silico* analyses predicting of folding structures induced by the rs114020893 T>C change in *NEXN-AS1*. (**A**) Corresponding to rs114020893T allele and (**B**) Corresponding to rs114020893C allele.

**15,900 lncRNAs from GENCODE (release 22)**

**690,564 SNPs**

**(MAF > 0.05)**

**Meta-analysis**

**using six TRICL GWASs**

**SNPs located at 15,531 autosomal lncRNAs with minor allele frequency (MAF)> 0.05**

**59 SNPs**

**(*P*< 1.00^-7^)**

**12,160 cases and 16,838 controls Additive genetic model**

**5 SNPs in chromosome 15 (reported)**

**53 SNPs in chromosome 6 (reported)**

**1 SNP in chromosome 1**

**Additional GWAS datasets of Harvard (984 cases and 970 controls) and deCODE (4009 cases and 221529 controls)**

**Focus on rs114020893 in lncRNA*NEXN-AS1* (Chr 1)**

**rs114020893 at 1p31.1as a new lung cancer risk-related locus**

**Supplementary Figure 3**. Flow-chart of SNP selection among the lncRNA genes
